# MoS_2_–NiO nanocomposite for H_2_S sensing at room temperature†

**DOI:** 10.1039/d3ra05241a

**Published:** 2023-09-29

**Authors:** Shama Sadaf, Hongpeng Zhang, Ali Akhtar

**Affiliations:** a Marine Engineering College, Dalian Maritime University Dalian 116026 China zhppeter@dlmu.edu.cn +86 411 84729934; b School of Information Science and Technology, Dalian Maritime University Dalian 116026 Liaoning P. R. China

## Abstract

The layered 2-D materials, such as molybdenum disulfide (MoS_2_), are among the most promising candidates for detecting H_2_S gas at very low concentrations. Herein, we have designed a series of novel nanocomposites consisting of MoS_2_ and NiO. These materials were synthesized *via* a simple hydrothermal method. The microstructure and morphology of nanocomposites were studied using different characterization techniques, such as X-ray diffraction (XRD), scanning electron microscopy (SEM), transmission electron microscopy (TEM), high-resolution transmission electron microscopy (HRTEM), Brunauer–Emmett–Teller (BET) analysis, and X-ray photoelectron spectroscopy (XPS). These nanocomposites were used as gas sensors, and the highest response (6.3) towards 10 ppm H_2_S was detected by the MNO-10 gas sensor among all the tested sensors. The response value (*R*_g_/*R*_a_) was almost three times that of pure NiO (*R*_g_/*R*_a_ = 2). Besides, the MNO-10 sensor exposed good selectivity, short response/recovery time (50/20 s), long-term stability (28 days), reproducibility (6 cycles), and a low detection limit (2 ppm) towards H_2_S gas at RT. The excellent performance of MNO-10 may be attributed to some features of MoS_2_, such as a layered structure, higher BET surface area, higher active sites, and a synergistic effect between MoS_2_ and NiO. This simple fabrication sensor throws a novel idea for detecting H_2_S gas.

## Abstract 2

The layered 2-D materials, such as molybdenum disulfide (MoS_2_), are among the most promising candidates for detecting H_2_S gas at very low concentrations.

## Graphical abstract

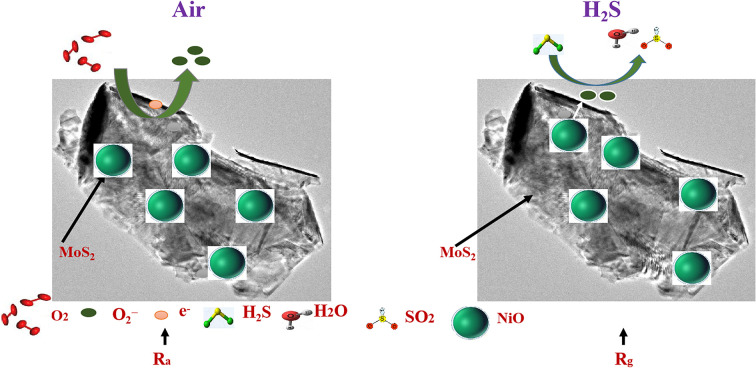

## 1. Introduction

Detecting hazardous and flammable volatile organic compounds (VOCs), such as hydrogen sulfide (H_2_S), having rotten eggs smell is important for protecting human health. The excessive inhalation of H_2_S, around 250 ppm, may cause the death of human beings.^1^ A very low concentration of H_2_S can cause various chronic diseases, such as poor memory, throat injury, dizziness, cough, and damage to the human nervous system.^2^ In this regard, H_2_S gas sensors with high response and low limit of detection (LOD) are highly required.

Semiconductor metal oxides (SMOs) are highly recommended and studied as gas sensors because of their advantages of low cost, simple fabrication, and ability to synthesize various nanocomposites.^3^ Up to now, different p-type SMOs, such as nickel oxide (NiO), tin oxide (SnO_2_), nickel cobaltite (NiCo_2_O_4_), zinc cobaltite (ZnCo_2_O_4_), and zinc oxide (ZnO), have received wide attention in the field of gas sensors^4–8^ and supercapacitors.^9^ Among these SMOs, cubic NiO, having a band-gap of 3.5 eV, can be used to design novel nanocomposites with different metal oxides due to its marvelous properties, such as electrical, chemical, and thermal stabilities. NiO was found to be a promising candidate for detecting various gases, such as ozone,^10^ acetone,^11^ H_2_S,^12^ NH_3_,^13^ and formaldehyde.^14^ However, the reported NiO gas sensors still need improvement due to some demerits, such as high temperature, low response, and poor selectivity. Designing novel nanocomposites of NiO with layered 2-D materials is crucial to achieving these objectives.

MoS_2_, an n-type semiconductor having a band-gap of 1.29 eV, is one of the most well-known 2-D transition material dichalcogenides, and especially the layered structures of 2-D materials have received considerable attention because of their strong adsorption, high reactivity, larger surface area to volume ratio, and good electrical conductivity.^15^ Except for the gas sensors, it has been used in many fields, such as photo-catalysis,^16^ supercapacitors,^17^ and lithium-ion batteries.^18^ Other 2-D materials also find novel applications as gas sensors^19–22^ and supercapacitors.^23^ However, MoS_2_ can easily be composited with various SMOs to design novel electronic devices; these features make MoS_2_ a very promising candidate for detecting hazardous VOCs at low concentrations. In particular, Bai *et al.* proposed a sensor based on the hetero-structure of MoS_2_/SnO_2_, which exposed the gas sensing properties towards NO_2_ at room temperature. The optimized sensor showed a high response, short response/recovery time, good stability, and selectivity. Besides, many sensors, such as MoS_2_/SnO_2_ (CO sensor),^24^ CuO/MoS_2_ (NO_2_ sensor),^25^ MoS_2/_ZnO (NO_2_ sensor),^26^ Au/MoS_2_ (NO_2_ sensor),^27^ MoS_2_/ZnO–Zn_2_SnO_4_ (H_2_S sensor),^28^ and PtO_2_/MoS_2_ (NH_3_ sensor),^29^ have been exploited to investigate sub-ppm level gases.

Herein, a series of novel nanocomposites based on NiO spherical nanoparticles and layered MoS_2_ were designed for detecting toxic gases. Numerous characterizations such as XRD, SEM, TEM, HRTEM, BET, and XPS were performed for these nanocomposites. The gas sensing properties of the proposed MNO-10 sensor suggested a high response, good selectivity, short response/recovery time, and reliable long-term stability towards 10 ppm H_2_S among all other tested sensors (MNO-0, MNO-5, and MNO-15).

## 2. Experimental section

### 2.1 Materials

All the chemicals used in the synthesis method were bought from Sinopharm Chemical Reagent Co., Ltd. (Shanghai, China). The materials such as molybdenum disulfide (MoS_2_), nickel chloride hexahydrate (NiCl_2_·6H_2_O), and sodium hydroxide (NaOH) were utilized in the synthesis method without further purification.

### 2.2 Synthesis of NiO spherical nanoparticles and nanocomposites of MoS_2_–NiO

**Fig. 1:**
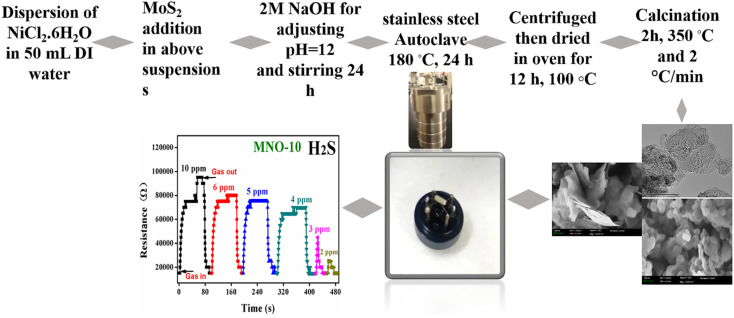
Synthesis diagram of MoS_2_–NiO nanocomposites.

NiO spherical nanoparticles and nanocomposites with different contents of MoS_2_ were subsequently synthesized *via* a simple hydrothermal method, and the details are expressed in Fig. 1. Initially, NiCl_2_·6H_2_O (1.5 g) was mixed with 50 mL deionized water (DI) in four different beakers; after half an hour of stirring, various contents of MoS_2_ such as 0.00 g, 0.0505 g, 0.101 g, and 0.151 g were dispersed in all four suspensions. These samples were named MNO-0, MNO-5, MNO-10, and MNO-15, respectively. After that, 2 M NaOH was added to adjust pH = 12. The samples were stirred for 24 h, then the next day, the mixtures were settled in 50 mL stainless steel autoclaves, and the oven was set for 24 h at the operating temperature of 180 °C. The obtained products were washed with DI and ethanol three times using centrifugation. After drying (12 h, 100 °C), the products were calcined at the operating temperature of 350 °C for 2 h and 2 °C min^−1^. The dried samples, after calcination, were ground in a mortar for different characterizations such as XRD, SEM, and TEM. All these tests were performed by providing various amounts of samples, such as 20–30 mg, 10 mg, 10 mg, 200 mg, and 5–20 mg powder, for XRD, SEM, TEM, BET, and XPS, respectively.

### 2.3 Fabrication of a sensor

The gas sensor diagram is shown in Fig. 1. The fabrication of sensors was reported in a previous work.^6^ After calcination, the paste to fabricate the sensor was prepared using 0.1 g powder and 2 drops of terpineol (the volume was approximately 0.1–0.2 mL for each drop of terpineol) and ground in a mortar. After that, the paste was coated onto the outer surface of an alumina tube with a small brush and then heated the alumina tube in an oven for 2 h at 80 °C. The purpose of the Ni–Cr heating wire was to control the operating temperature. Pt wires were given to link the alumina tube to the gas sensor device. All the hazardous gases detected in the present work were bought from Dalian Haide Technology Company Limited. NiO is a p-type material, and the response was calculated, such as the ratio of gas sensor resistance in gas (*R*_g_) to that of the resistance in air (*R*_a_) (*S* = *R*_g_/*R*_a_). Other important parameters, such as selectivity, stability, reproducibility, limit of detection (LOD), and response/recovery time, are discussed in this paper.

### 2.4 Physical characterization of materials

The micro-structural properties and morphologies of the synthesized products were observed by X-ray diffraction (XRD, D/MAX-Ultima, Cu Kα source, 2° min^−1^ scanning rate and the scanning angle from 10° to 80° as well as the power was 40 kV and 40 mA, Rigaku, Tokyo, Japan), scanning electron microscopy (SEM, ZEISS Gemini 500, Carl Zeiss AG, Oberkochen, Germany), transmission electron microscopy (TEM, JEM-3200FS, JEOL, Tokyo, Japan), high-resolution transmission electron microscopy (HRTEM, JEM-2100F, JEOL, Tokyo, Japan). Besides, X-ray photoelectron spectroscopy and Brunauer–Emmett–Teller analysis were carried out using XPS, ESCALAB 250XI, Thermo Fisher Scientific, Waltham, MA, USA and BET, ASAP2010C instrument, Norcross GA, USA, respectively.

## 3. Experimental results and discussion

### 3.1 Morphology and structure

**Fig. 2:**
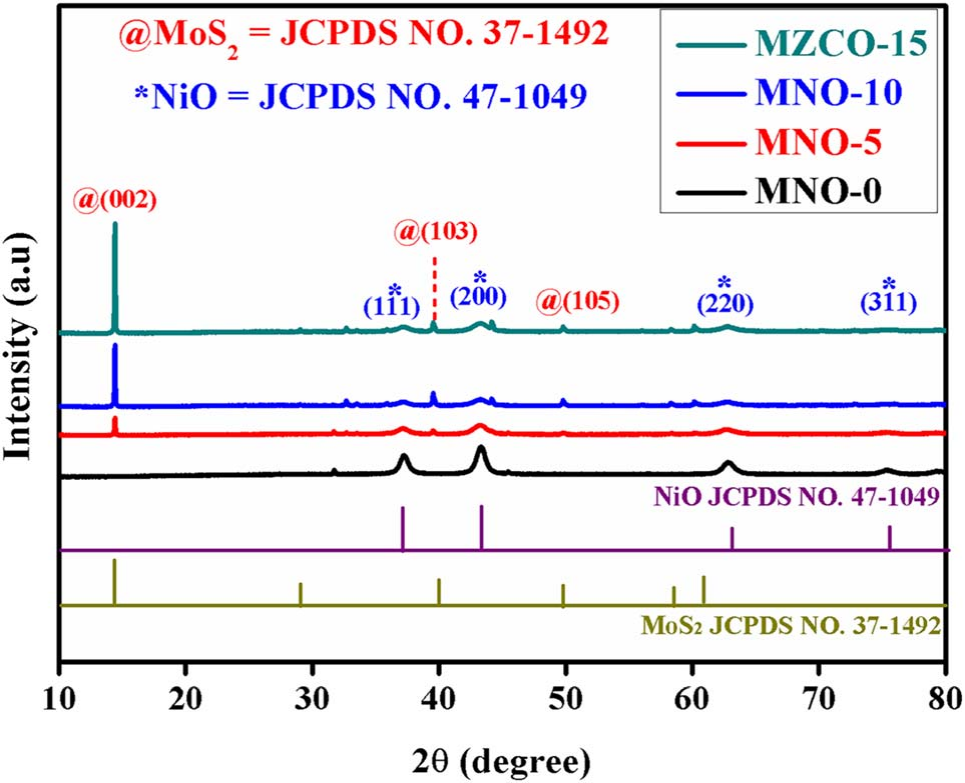
XRD patterns of MoS_2_–NiO nanocomposites.

The crystalline nature of the synthesized products was observed using XRD diffraction peaks, as exposed in Fig. 2. The diffraction peaks observed from the patterns corresponded to the crystallographic standard database files of MoS_2_ and NiO. The XRD diffraction patterns of all the samples showed the diffraction peaks of NiO at the 2*θ* values of 37.24°, 43.27°, 62.87°, and 75.41°, corresponding to (111), (200), (220), and (311) planes of NiO (JCPDS no. 47-1049), respectively. Except for the diffraction peaks of MNO-0, one high-intensity MoS_2_ peak and two small peaks were observed in all other nanocomposites at 2*θ* values of 14.37°, 39.53°, and 49.78°, which matched with (002), (103) and (105) planes of MoS_2_ (JCPDS no. 37-1492). No extra peaks were found in all patterns, suggesting that the samples were well-ordered and possessed good crystallinity and clarity. Besides, crystallite sizes of NiO spherical nanoparticles in all nanocomposites were calculated using the Debye–Scherrer formula, yielding 15.59, 10.86, 9.36, and 10.07 nm based on the (200) peaks, while 14.02, 10.23, 9.98, and 9.39 nm based on the (111) peak in the nanocomposites of MNO-0, MNO-5, MNO-10, and MNO-15, respectively. It was found that adding MoS_2_ in the nanocomposites decreased the crystallite size of NiO.

**Fig. 3:**
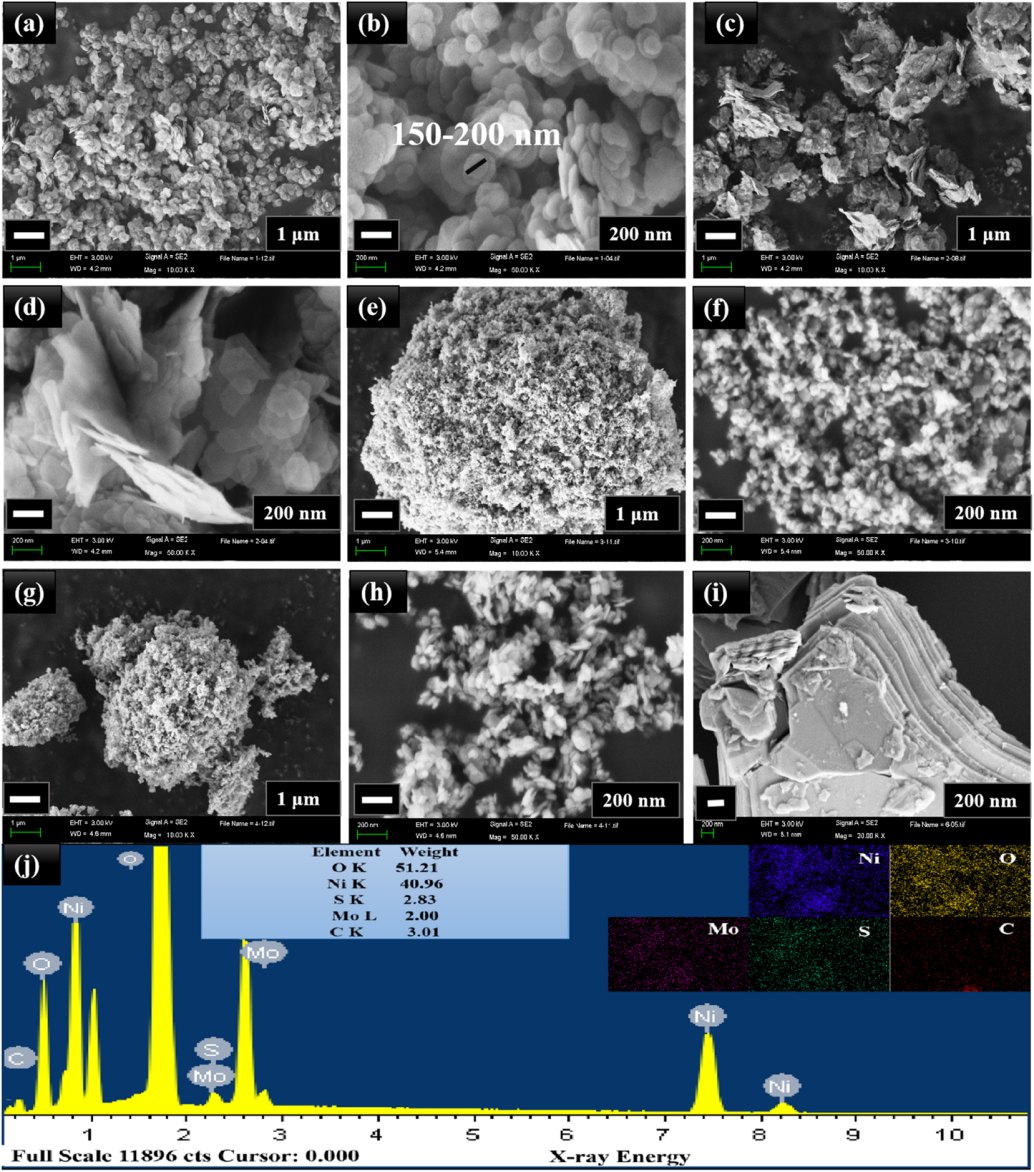
(a and b) SEM images of MNO-0, (c and d) MNO-5, (e and f) MNO-10, (g and h) MNO-15, and (i) MoS_2_. (j) EDS spectrum and mappings of MNO-10.

Fig. 3 reveals the SEM images of all the nanocomposites. The spherical NiO nanoparticles with an average particle size of 150–200 nm are seen from the SEM images of MNO-0 in Fig. 3(a) and (b). The SEM images of the nanocomposite MNO-5 in Fig. 3(c) and (d) and SEM images of the nanocomposite MNO-10 and MNO-15 are displayed in Fig. 3(e)–(h), respectively. Fig. 3(i) shows the SEM image of MoS_2_ displaying the layered structure. From SEM images, first of all, the morphology of layered MoS_2_ and spherical nanoparticles NiO were observed; secondly, it was noticed that the particle size of NiO was reduced by adding MoS_2_ in MNO-5, MNO-10, and MNO-15 nanocomposites. SEM results corresponded to XRD results, which stated that by adding MoS_2_ to the samples, the crystallite sizes of NiO were reduced. The uniform scattering of all the elements present in MNO-10 and EDS spectrum is disclosed in Fig. 3(j).

**Fig. 4:**
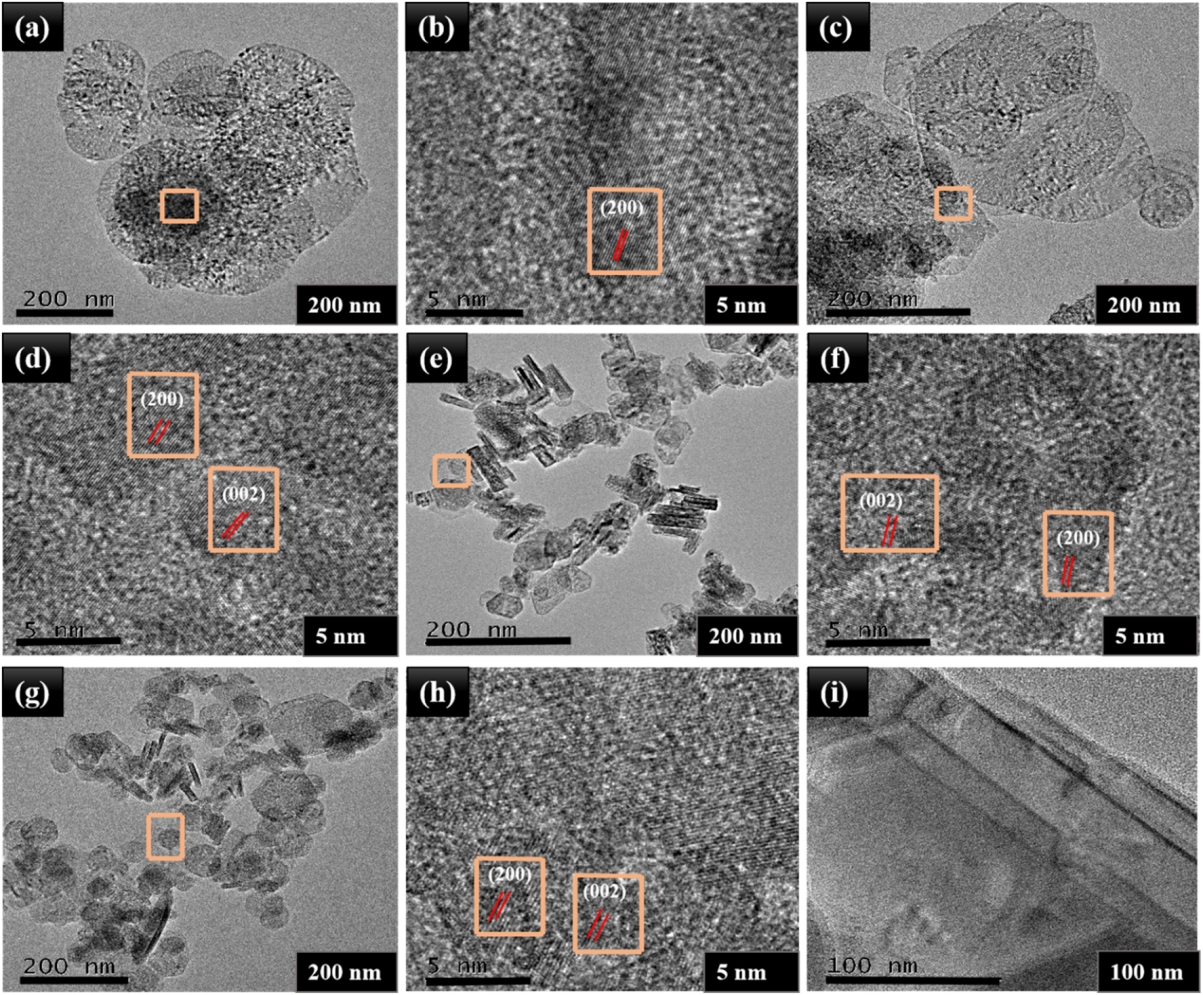
TEM images and HRTEM images of (a and b) MNO-0, (c and d) MNO-5, (e and f) MNO-10, (g and h) MNO-15, and (i) MoS_2_.

The decoration of NiO spherical nanoparticles onto the layered structure of MoS_2_ was further verified by TEM and HRTEM images. In Fig. 4(a) and (b), TEM images of MNO-0 are disclosed, which showed the average particle size (150–200 nm) of spherical NiO nanoparticles; besides, the clear lattice fringes with a lattice spacing of *d* = 0.21 nm corresponded to the (200) plane of NiO nanoparticles. Also, in Fig. 4(c)–(h), TEM images proved the layered structure of MoS_2_ and spherical nanoparticles of NiO, and HRTEM images exposed lattice spacing's of *d* = 0.62 and *d* = 0.21 nm related to the (002) and (311) planes of MoS_2_ and NiO nanoparticles. In Fig. 4(i), the layered structure of MoS_2_ was verified by TEM analysis. TEM and HRTEM revealed the particle sizes of NiO (150–200 nm). Gradually, their size was reduced by adding MoS_2_ contents in the nanocomposite, corresponding to the XRD results.

**Fig. 5:**
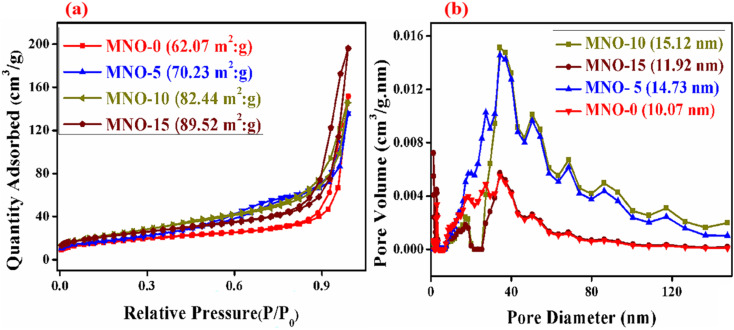
N_2_ adsorption–desorption isotherms and pore size distributions of MNO-0, MNO-5, MNO-10, and MNO-15 (a and b).

In Fig. 5, the nitrogen adsorption–desorption isotherms were analyzed for the MNO-0, MNO-5, MNO-10, and MNO-15 nanocomposites to interpret their porosity and distribution. Nitrogen adsorption–desorption isotherms reveal the specific surface area of all the nanocomposites. A higher BET surface area corresponds to a higher gas-sensing response. BET surface areas of nanocomposites of MNO-0, MNO-5, MNO-10, and MNO-15, as shown in Fig. 5(a), were 62.07 m^2^ g^−1^, 70.23 m^2^ g^−1^, 82.44 m^2^ g^−1^ and 89.52 m^2^ g^−1^, respectively. Besides, in the images of Fig. 5(b), the pore size distribution diagrams are seen, suggesting that all the nanocomposites have an obvious H3 hysteresis loop, which verified the presence of many pores as well as the mesoporous nature of samples.^30^ It was found that pore volumes and pore sizes of MNO-0, MNO-5, MNO-10, and MNO-15 were 0.2346 cm^3^ g^−1^, 0.2093 cm^3^ g^−1^, 0.3034 cm^3^ g^−1^, 0.2254 cm^3^ g^−1^, and 15.12 nm, 11.92 nm, 14.73 nm, 10.07 nm, respectively. Due to the higher BET surface areas and wide pore size distributions of nanocomposites, these were denoted as high gas sensing responsive, especially the gas sensor of MNO-10, which detected high response towards 10 ppm H_2_S.

**Fig. 6:**
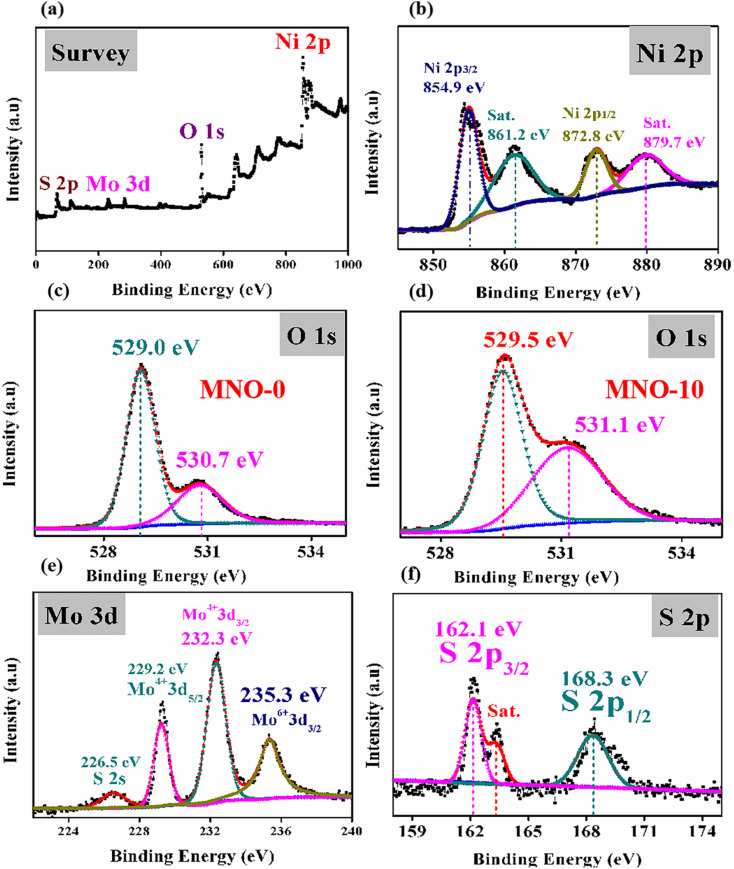
XPS spectra (a) full survey of MNO-10 (b) Ni 2p spectrum of MNO-10, (c) O 1s spectrum of MNO-0, (d) O 1s spectrum of MNO-10, (e) Mo 3d spectrum of MNO-10, (f) S 2p spectrum of MNO-10.

The surface chemical composition and electronic state of MNO-0 and MNO-10 nanocomposites were measured using X-ray photoelectron spectroscopy. The details are as follows: in Fig. 6(a), the full scan XPS spectrum is given, which specifies the presence of elements such as Ni, O, M, and S in the MNO-10 nanocomposite. Next, the high-resolution spectrum of Ni 2p is displayed in Fig. 6(b). The two peaks in the spectrum of MNO-10 at 854.9 eV and 872.8 eV corresponded to Ni 2p_3/2_ and Ni 2p_1/2_,^31,32^ along with two satellite peaks (861.2 eV and 879.7 eV). O 1s spectra in Fig. 6(c) and (d) revealed two peaks in both MNO-0 and MNO-10 nanocomposites at 529.0, 530.7 eV and 529.5, 531.1 eV, and these peaks were related to two oxygen states such as the crystal lattice (O_latt._) and chemisorbed oxygen specie (O_ads._), respectively.^33^ In Fig. 6(e), Mo 3d spectrum of MNO-10 is displayed, and four peaks at 226.5 eV, 229.2 eV, 232.3 eV, and 235.2 eV related to S 2s, Mo^4+^ 3d_5/2_, Mo^4+^ 3d_3/2_ and Mo^6+^ 3d_5/2_.^34,35^ In Fig. 6(f), the S 2p spectrum of MNO-10 shows two peaks at 162.1 and 168.3 eV, corresponding to S 2p_3/2_ and S 2p_1/2_.^36,37^ A satellite peak is also displayed in the spectra of S 2p.

### 3.2 Gas sensing performance

**Fig. 7:**
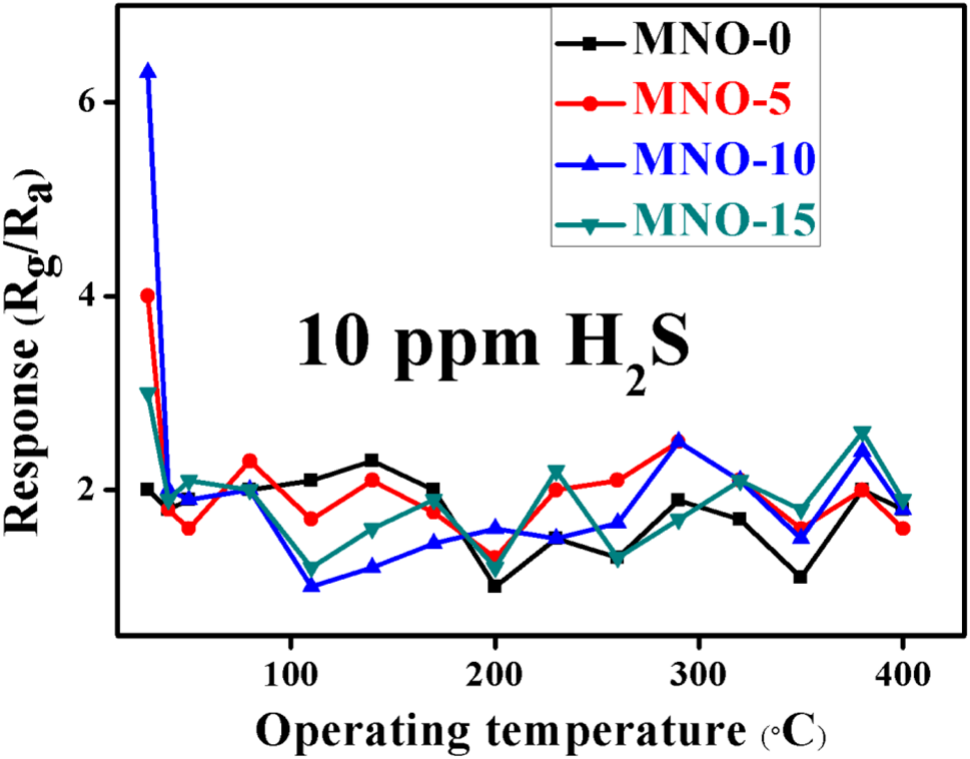
The responses of various gas sensors at different operating temperatures.

The operating temperature and selectivity are also one of the most important parameters for gas sensor applications. All the gas sensors based on various nanocomposites MNO-*P*, *P* = 0, 5, 10, and 15 showed responses towards 10 ppm H_2_S at different temperatures, as shown in Fig. 7. The results proved that the sensors showed the highest responses at room temperature. The sensor of MNO-10 displayed the highest response towards 10 ppm H_2_S among all other tested sensors and the response (*R*_g_/*R*_a_) was 6.3, which was 3.15 times that of pure NiO spherical nanoparticles. The results also verified that the response was greatly affected by the operating temperature. When the temperature was increased, the electrons from the semiconductor material surface were likely to break down from the nucleus of the material and form free electrons, which in turn increased the free electron concentration and decreased the resistance as well as the response of the sensor.^3^

**Fig. 8:**
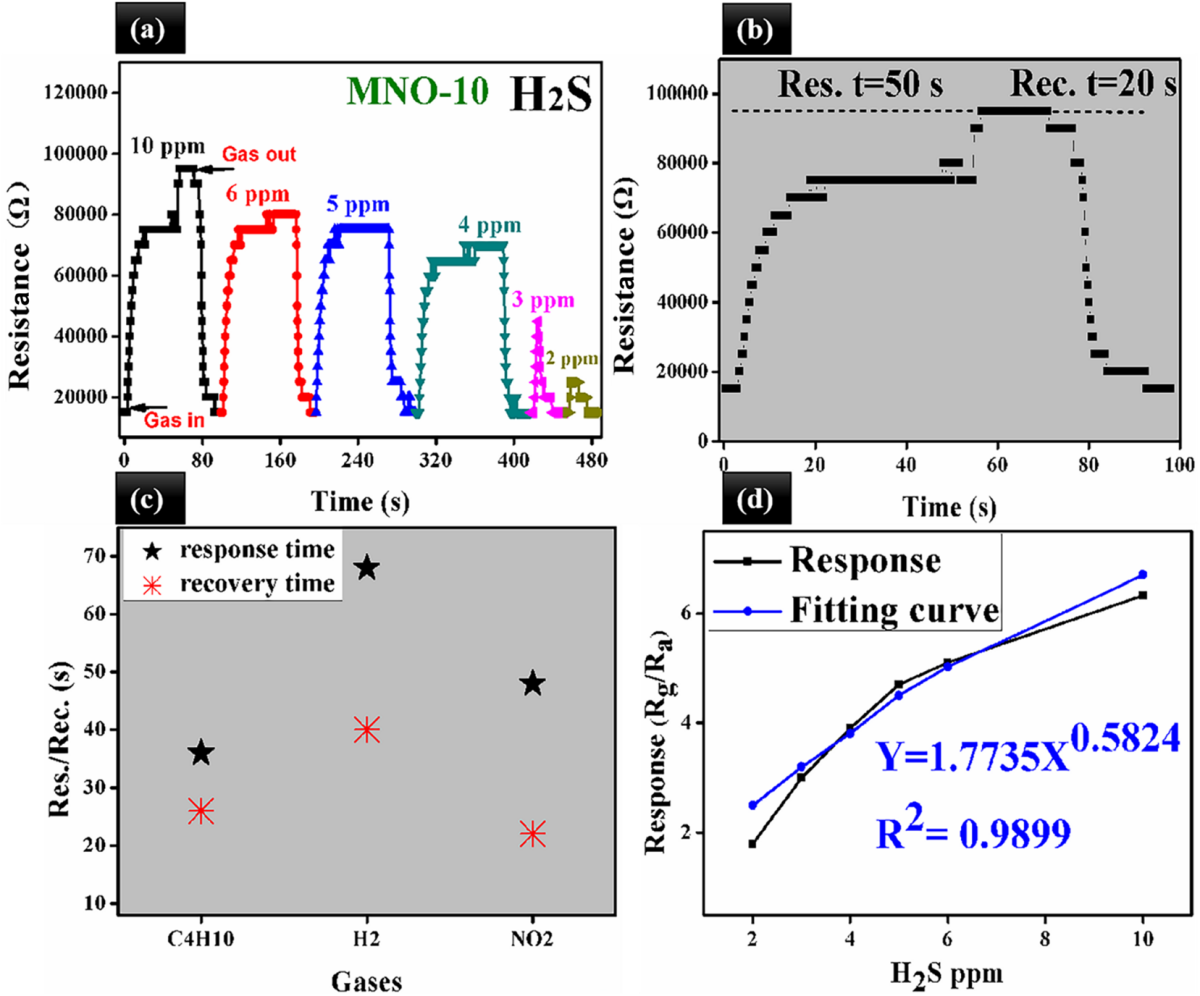
(a) Dynamic res./rec. curve and resistance of MNO-10 to 10–0.5 ppm H_2_S at RT, (b) resistance change of MNO-10 composite-based sensor to 10 ppm H_2_S, (c) the plots of response/recovery times towards 10 ppm C_4_H_10_, H_2_, and NO_2_ of MNO-10, (d) relation between response and different concentrations of H_2_S of MNO-10.

The dynamic response transients of MNO-10 towards various H_2_S concentrations are shown in Fig. 8(a). The response/recovery times for 10, 6, 5, 4, 3, and 2 ppm were 50/20 s, 45/18 s, 12/16 s, 7/8 s and 6/8 s, respectively. Besides, the response to 2 ppm H_2_S was 1.8. In order to spotlight the response/recovery speed towards 10 ppm H_2_S, we have placed a graph between resistance and res./rec. time in Fig. 8(b), which showed that when the sensor was in H_2_S atmosphere, the curve went higher (res. time, 50 s) and around (95 000 Ω) it was in the stable state, then in air atmosphere, it started to go back towards the original state (rec. time, 2 s). In Fig. 8(c), the res./rec. times of MNO-10 towards C_4_H_10_, H_2_, and NO_2_ are shown, with response times 36, 68, and 48 s, and recovery times 26, 40, and 22 for C_4_H_10_, H_2_, and NO_2_, respectively. The linear relation between the response and H_2_S ppm is crucial because linearity can make a sensor a promising candidate in gas sensors. Fig. 8(d) shows that the responses of the sensor (MNO-10) were 6.3, 5.1, 4.7, 3.9, 3, 1.9 towards 10, 6, 5, 4, 3, and 2 ppm, respectively, while fitting curve values were 6.7, 5.02, 4.5, 3.8, 3.2, and 2.5 based on the equation of *Y* = 1.7735*X*^0.5824^, and regression coefficient, *R*^2^ = 0.9899. It is clear from the figure that with increasing H_2_S concentrations, the response is gradually increased, suggesting its linearity between the response and H_2_S concentrations.

**Fig. 9:**
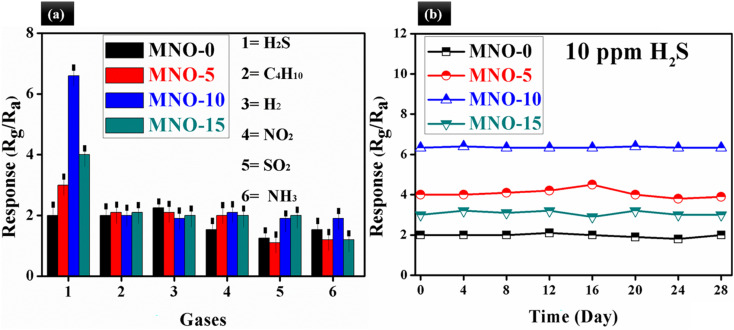
(a) The selectivity test for all sensors towards various gases at RT, (b) the stability test for all sensors at RT.

Selectivity is an essential parameter for a gas sensor. In our case, the selectivity of MZCO-*P*, *P* = 0, 5, 10 and 15, based gas sensors was measured, as shown in Fig. 9(a), and the selectivity was checked out using six toxic gases such as H_2_S, C_4_H_10_, H_2_, NO_2_, SO_2_, and NH_3_ (10 ppm) at RT. The sensor based on MNO-10 showed the response of 6.6, 2.0, 1.9, 2.1, 1.8 and 1.9 towards H_2_S, C_4_H_10_, H_2_, NO_2_, SO_2_ and NH_3_, respectively. The selectivity of MNO-10 was calculated, such as the ratio of the highest response towards H_2_S and the second highest response towards NO_2_ (selectivity = *S*_10 ppm H_2_S_/*S*__10_ ppm NO_2__), and its calculated selectivity was around 3. The selectivity of results stated that the response of MNO-10-based gas sensors towards 10 ppm H_2_S was almost three times higher than other checked VOCs, which pointed out that the sensor displayed a high response and impressive selectivity towards H_2_S. In Table 1, some sensors are compared against our sensor, which suggests that the current sensor exposed high response (6.3), short response/recovery times (50/20 s), good stability (28 days), better reproducibility (6 cycles), good selectivity (3.0), and linear relationship between response and H_2_S concentration. The above features revealed that the simple fabricated sensor in our work is a potential candidate for real-time applications. As far as we know, long-term stability and reproducibility are also imperative for gas sensors. Herein, Fig. 9(b) examines the stability test for all MZCO-*P*, *P* = 0, 3, 6, 9, and 12, nanocomposite-based gas sensors, which showed that the sensors were stable for 28 days. There was no obvious fluctuation in the response with time, exhibiting adequate stability for detecting H_2_S at RT. The most stable sensor was the MNO-10-based sensor among all other tested sensors.

**Table 1:** Comparison of various gas sensors^a^

Materials	Operating temp. (°C)	Res./rec. time	Response (gas ppm)	LOD	Stability	Ref.
PANI/MoS_2_/SnO_2_	RT	21 s/130 s	10.9 (NH_3_, 100 ppm)	0.2 ppm	—	40
SnO_2_/MoS_2_	RT	6.8 min/2.7 min	— (NO_2_, 10 ppm)	—	—	41
MoS_2_	130	1.4 s/2.9 s	86.9 (HCHO, 100 ppm)	10 ppm	30 days	42
NiO/SnO_2_	210	—	31.04 (HCHO, 50 ppm)	—	—	43
NiO/ZnO	200	18 s/30 s	9.3 (HCHO, 100 ppm)	—	—	44
NiO/ZnO	240	9 s/8 s	42 (HCHO, 100 ppm)	1 ppm	30 days	31
NiO/ZnO	140	31 s/49 s	142 (glycol, 100 ppm)	—	—	45
Au–MoS_2_	60	—	10 (NH_3_, 1000 ppm)	25 ppm	—	46
rGO/MoS_2_	RT	300 s/600 s	— (HCHO, 10 ppm)	—	—	47
NiCo_2_O_4_@SnO_2_	160	—	8.87 (ethanol, 100 ppm)	2 ppm	30 days	48
MoS_2_–NiO	RT	50 s/29 s	6.3 (H_2_S, 10 ppm)	2 ppm	28 days	This work

a Temp. = temperature, res./rec. = response/recovery. Ref. = references.

**Fig. 10:**
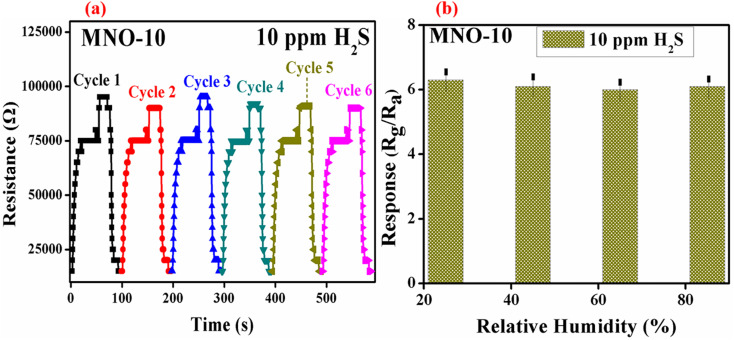
(a) The reproducibility of MNO-10 based gas sensor at RT towards 10 ppm H_2_S, (b) the response *vs.* different RH at RT towards 10 ppm H_2_S.

For detecting low concentrations of VOCs at RT, high selectivity and good reproducibility are imperative for a sensor to prove its promising applicability. Considering the above factors, reproducibility is also very important. Fig. 10(a) shows the six cycles of the gas sensing performance of MNO-10. As it is clear from the figure, the resistance in gas and air atmosphere was almost the same in six cycles; after every cycle, the resistance can go up in the H_2_S atmosphere and back into the air atmosphere, suggesting good reproducibility and reversibility of the current sensor. Fig. 10(b) shows the graph between the response of a sensor based on MNO-10 nanocomposite and relative humidity (RH). The sensor was tested at RT, and the response was decreased slightly at RH values of 45, 65, and 85, which verified that the sensor has impressive stability against humidity.

### 3.3 Gas sensing mechanism

**Fig. 11:**
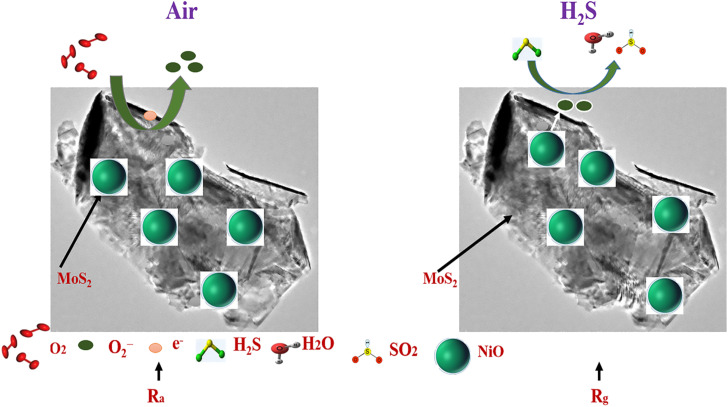
The gas sensing mechanism and energy band-gap structure of MNO-10 based gas sensor at RT towards 10 ppm H_2_S.

We have synthesized a series of nanocomposites based on layered MoS_2_ and spherical NiO nanoparticles and applied them as gas sensors, and the highest response towards 10 ppm H_2_S was received. For a deeper understanding, the gas sensing mechanism of nanocomposites should be studied, which is required for RT H_2_S detection. In general, the gas sensing mechanism is based on the variation of resistance in air and gas environments;^38^ however, resistance changes based on the charge carrier concentration of the material surface. In Fig. 11, the gas sensing mechanism and band diagram are described to prove the sensing mechanism. In our experiments, as we can see, the main sample, MNO-10, shows the highest response towards 10 ppm H_2_S compared with other tested sensors. The highest response may be correlated to some factors explained as follows: first of all, a p–n hetero-junction is formed between the n-type layered MoS_2_ and p-type NiO, which provides a large quantity of electron transfer in the nanostructures; secondly, the layered structure can facilitate the rapid diffusion and electrons transmission density (SEM); third, the layered structure of MoS_2_ enhanced the BET surface area of the nanocomposites, which can be considered one of the imperative factors to enhance the gas sensing properties such as high response, good selectivity, and short response/recovery times towards H_2_S (BET); fourth, moreover, as XPS approved it, more oxygen species can be adsorbed on the surface of the sensing material, which may help to increase the resistance.

Moreover, as shown in the diagram, when the sensor of MNO-10 was in an air atmosphere, the oxygen molecules were adsorbed on the surface of the material and accepted electrons to convert them into O_2_^−^ (adsorbed oxygen),^39^ besides making a hole accumulation layer (HAL) from the holes which are left on the surface, signifying the reduction of resistance. The reactions based on temperatures are described in eqn (1)–(4).

1 O_2(gas)_ → O_2(ads.)_,

2 O_2(ads.)_ + e^−^ → O_2(ads.)_^−^, *T* < 100 °C

3 O_2(ads.)_^−^ + e^−^ → 2O_(ads.)_^−^, 100 °C ≤ *T* ≤ 300 °C

4 O_(ads.)_^−^ + e^−^ → O_(ads.)_^2−^, *T* > 300 °C

After that, when the sensor of MNO-10 was in the H_2_S atmosphere, the sensor, herein, NiO is a p-type semiconductor. When the sensor was in the H_2_S atmosphere, it reacted with O_2_^−^ by forming SO_2_ and H_2_O, as shown in eqn (5); besides, there was an interaction between the H_2_S gas and hydroxyl species of the thin film sensor as well. During the reaction, the electrons returned to the conduction band to recombine holes simultaneously, which decreased the thickness of HAL, increasing the material's resistance. This would improve the gas sensing properties of the current sensor, and MNO-10 showed a p-type behaviour.

5 2H_2_S (g) + 2O_2_^−^ (ads.) → 2SO_2_ + 2H_2_O + 3e^−^

## 4. Conclusions

In this study, we designed a new series of sensors based on the nanocomposite of layered MoS_2_ and spherical NiO nanoparticles; these nanocomposites were synthesized hydrothermally. Various characterizations were performed, for example, XRD, SEM, TEM, HRTEM, BET, and XPS, to confirm morphology and structural properties. From XRD, it was shown that the crystallite size was reduced with the increase of MoS_2_ contents in the nanocomposites, and particle size also decreased by adding MoS_2_ (SEM, TEM). Further, BET results proved that the nanocomposites have higher BET-specific surface areas than pure NiO and more oxygen adsorption of MNO-10 than pure NiO verified by XPS. After that, the synthesized materials were tested as gas sensors, and the gas sensor was fabricated through a mixture of solid and terpineol oil. Various sensors based on nanocomposites (MNO-*A*, *A* = 0, 5, 10, 15) were used for detecting four kinds of toxic gases. The highest response towards 10 ppm H_2_S gas was detected by the gas sensor of MNO-10 among all other tested sensors. The response was 6.3 for 10 ppm H_2_S. Besides the highest response, the sensor of MNO-10 exposed good selectivity (3.0), better stability (28 days), minimum LOD (2 ppm), good reproducibility (6 cycles), and an almost linear relationship between response and concentrations of H_2_S (2–10 ppm). The marvelous gas sensing properties of MNO-10 were related to some crucial points such as p–n hetero-junction, layered structure of MoS_2_, higher BET surface area, and increased adsorption of oxygen species. Room temperature H_2_S gas sensors fabricated through simple ways are finding novel applications in daily routine activities. In this way, our synthesized nanocomposite-based gas sensor has promising applicability.

## Conflicts of interest

This work does not have any conflict of interest.

## Supplementary Material

RA-013-D3RA05241A-s001
