# Supplementary material for: MoS2–NiO nanocomposite for H2S sensing at room temperature
Source: RSC Adv. 2023 Sep 29;13(41):28564–75. doi: 10.1039/d3ra05241a (PMC10539850; doi:10.1039/d3ra05241a)
Supplement: RA-013-D3RA05241A-s001 [file RA-013-D3RA05241A-s001.pdf]

Electronic Supplementary Material (ESI) for RSC Advances.
This journal is © The Royal Society of Chemistry 2023

SEM images of MNO-0

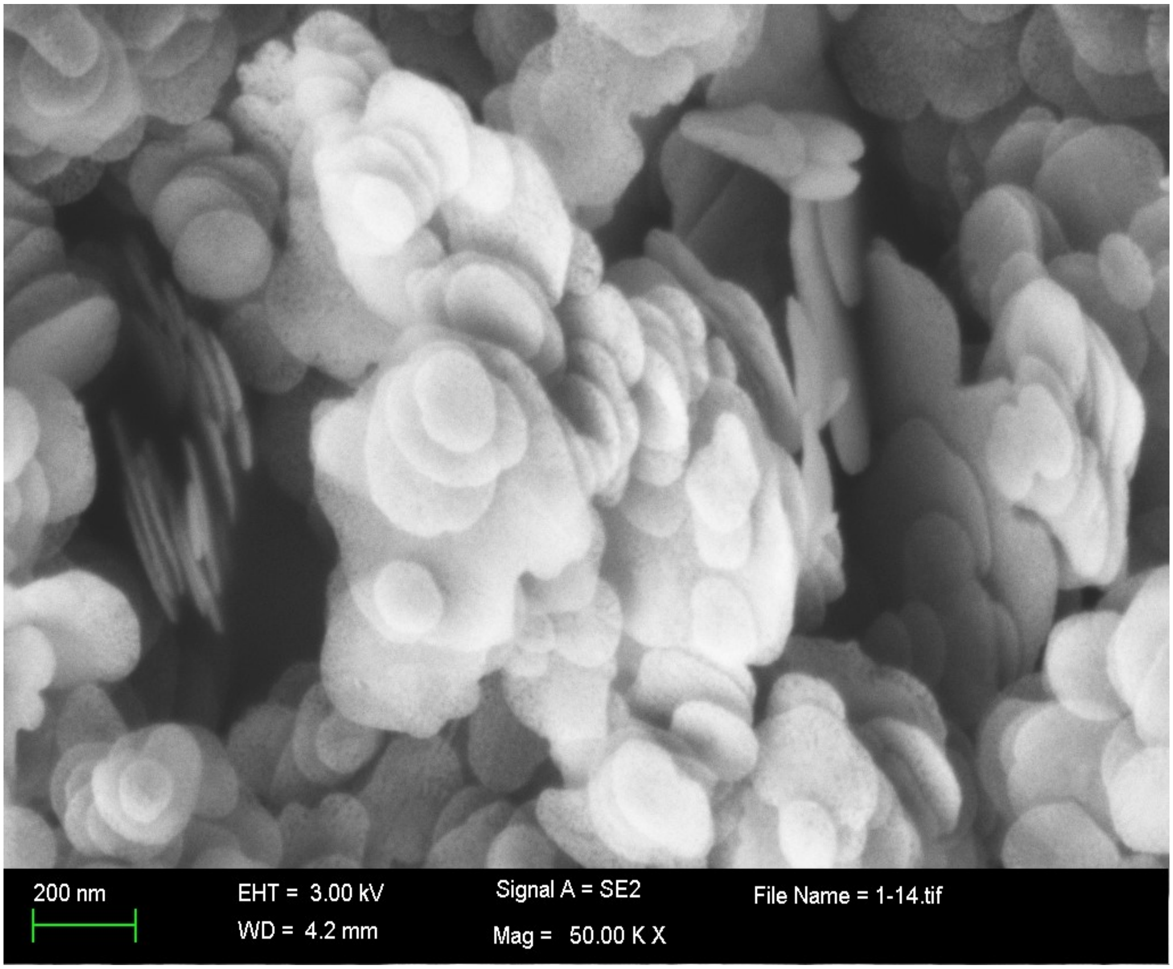

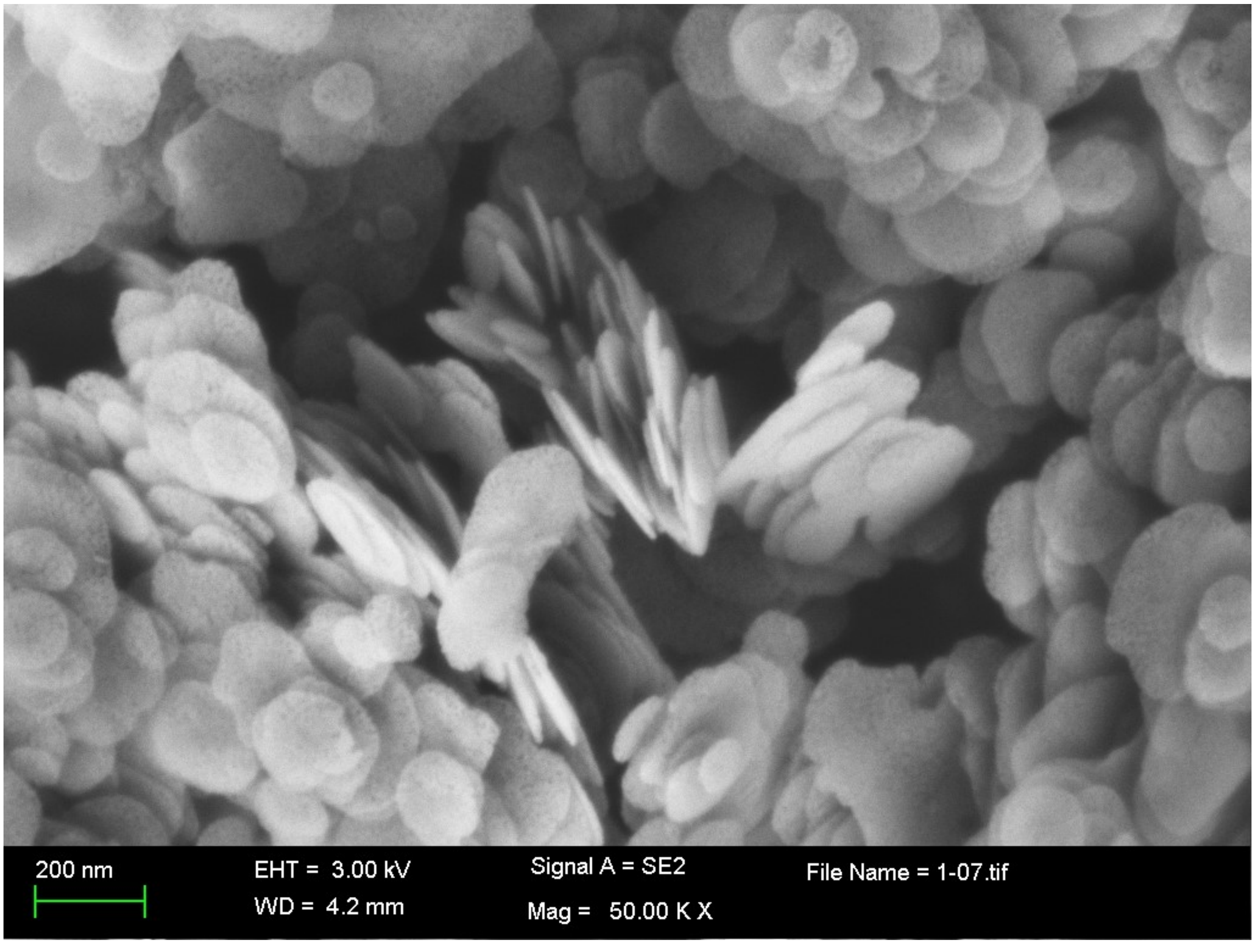

SEM images of MNO-5

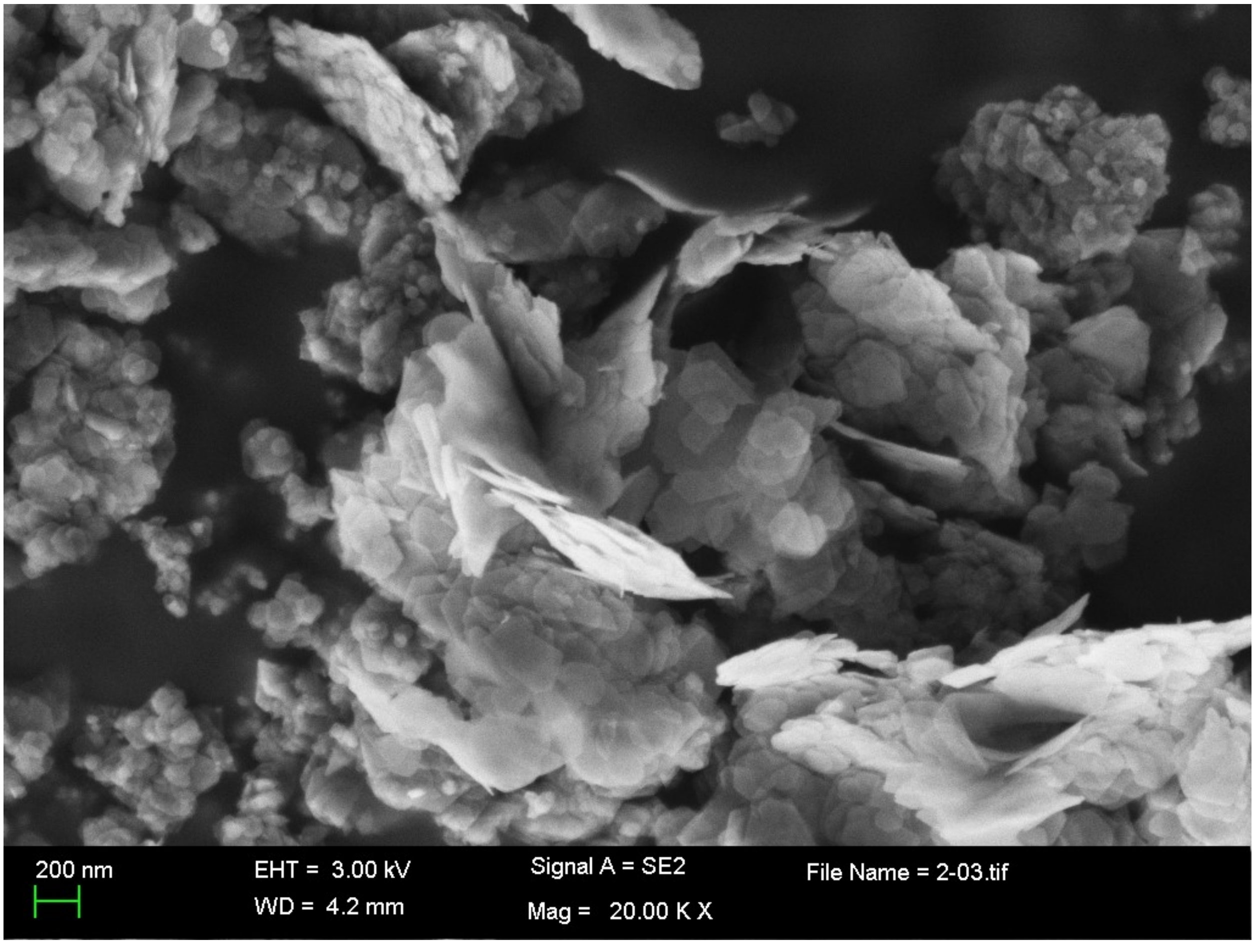
200 nm
EHT = 3.00 kV
WD = 4.2 mm
Signal A = SE2
Mag = 20.00 K X
File Name = 2-03.tif

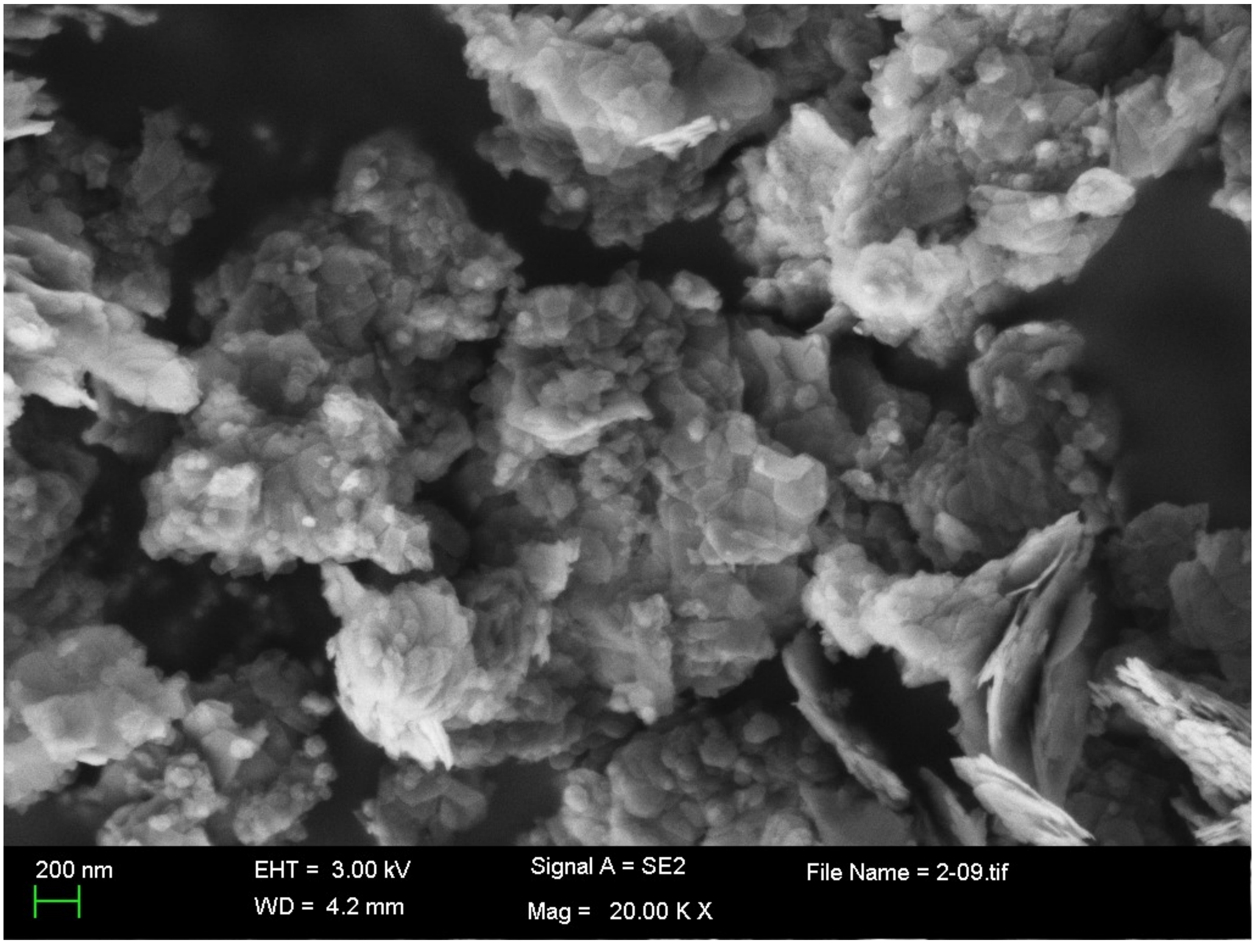

SEM images of MNO-10

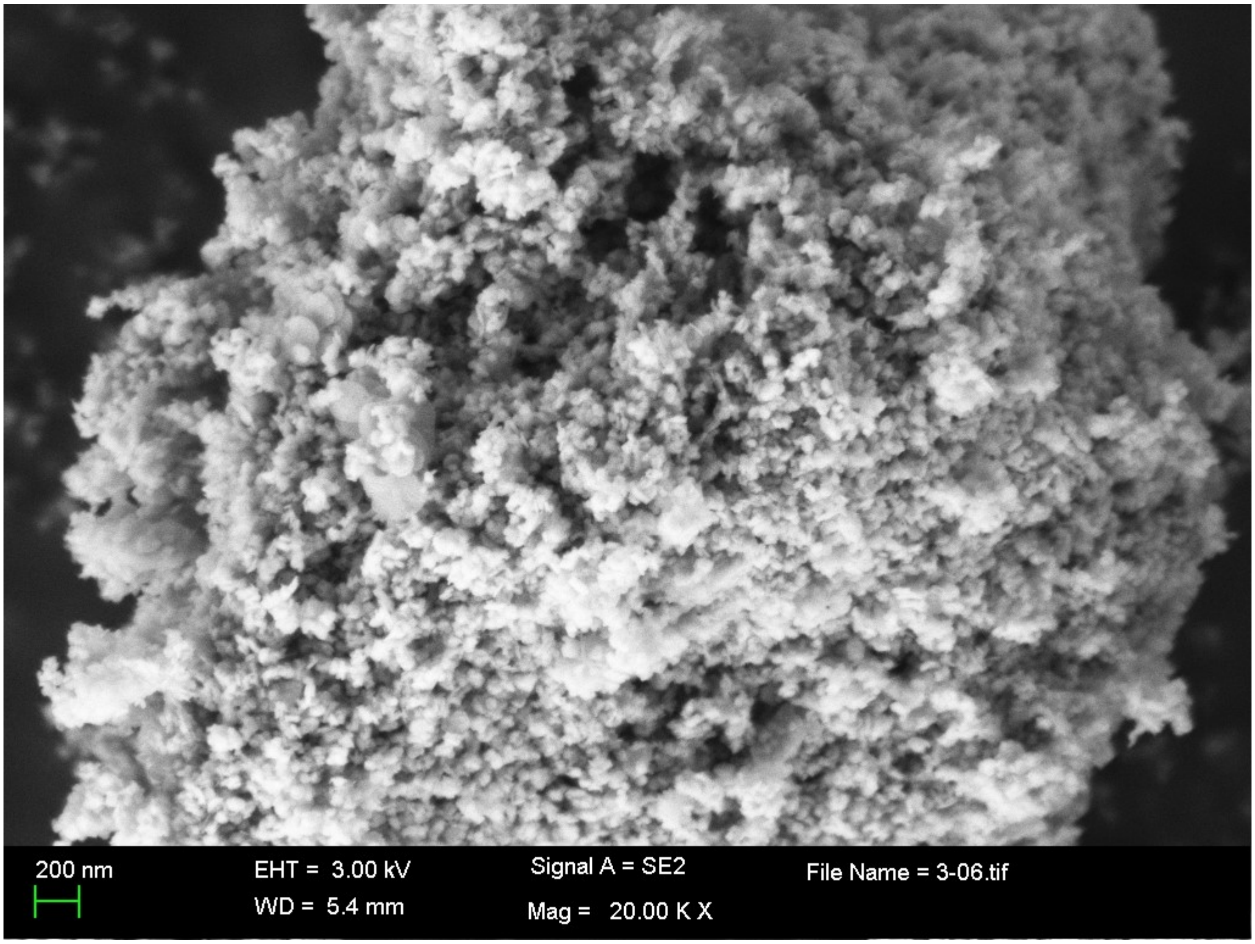
200 nm
EHT = 3.00 kV
WD = 5.4 mm
Signal A = SE2
Mag = 20.00 K X
File Name = 3-06.tif

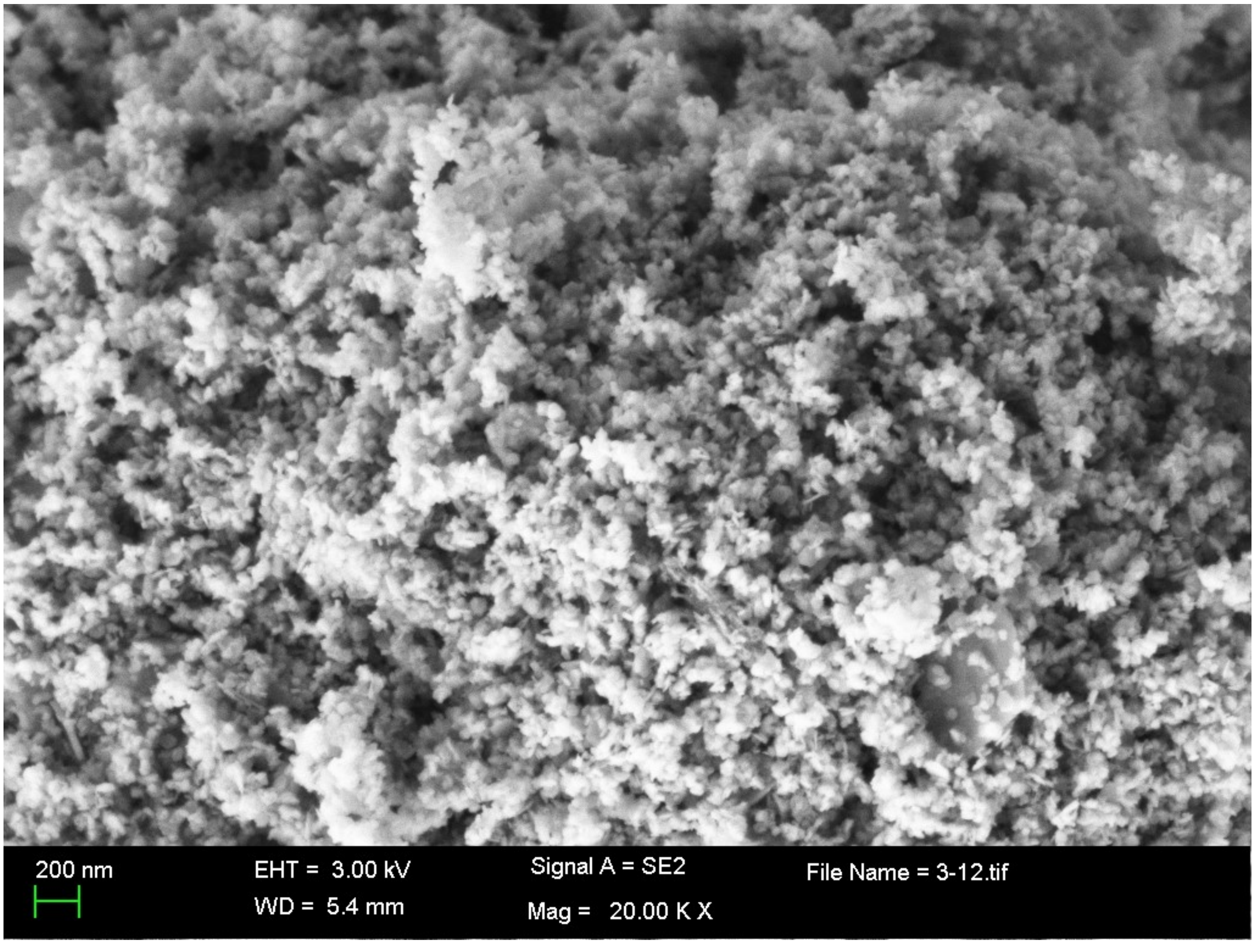

SEM images of MNO-15

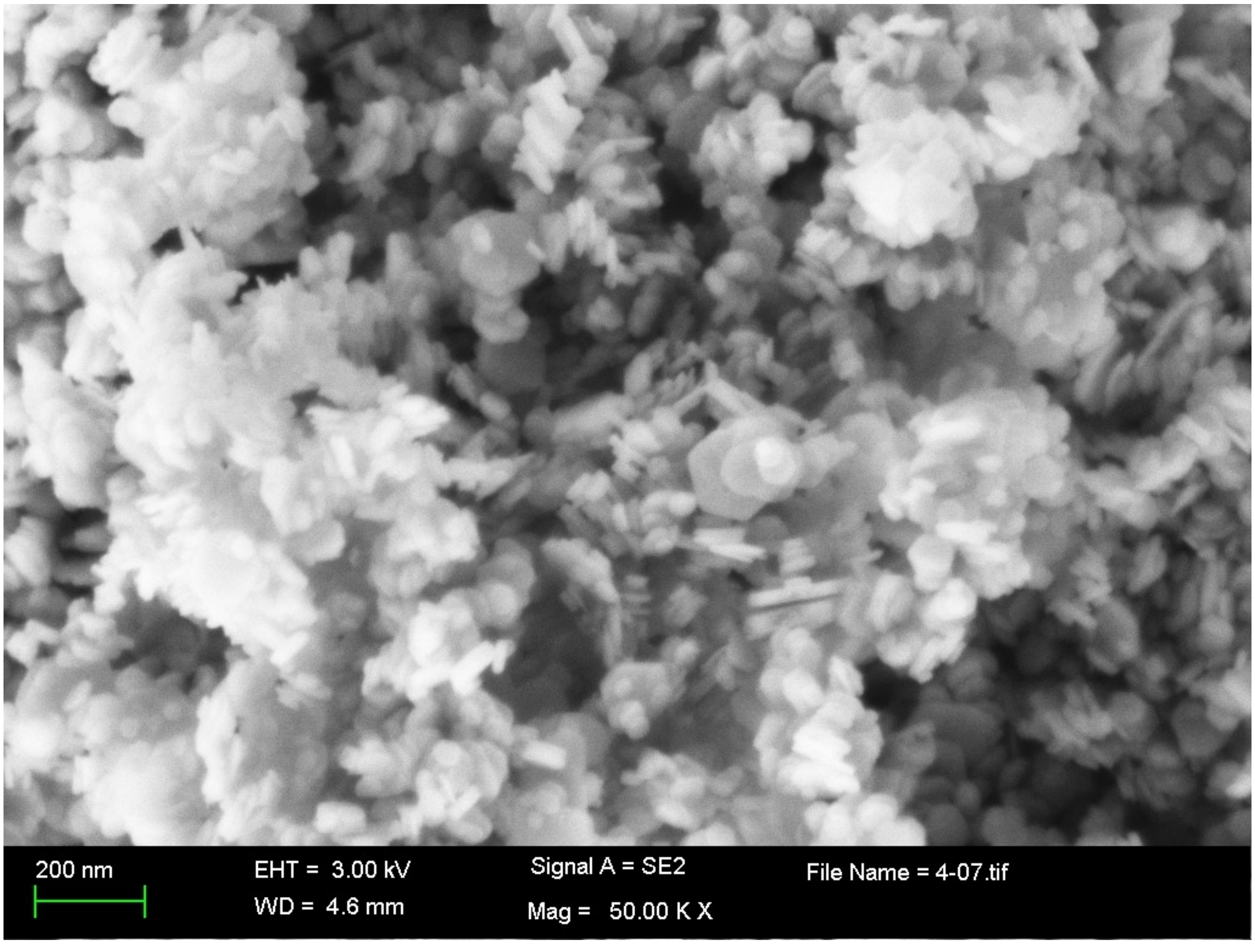
200 nm
EHT = 3.00 kV
WD = 4.6 mm
Signal A = SE2
Mag = 50.00 K X
File Name = 4-07.tif

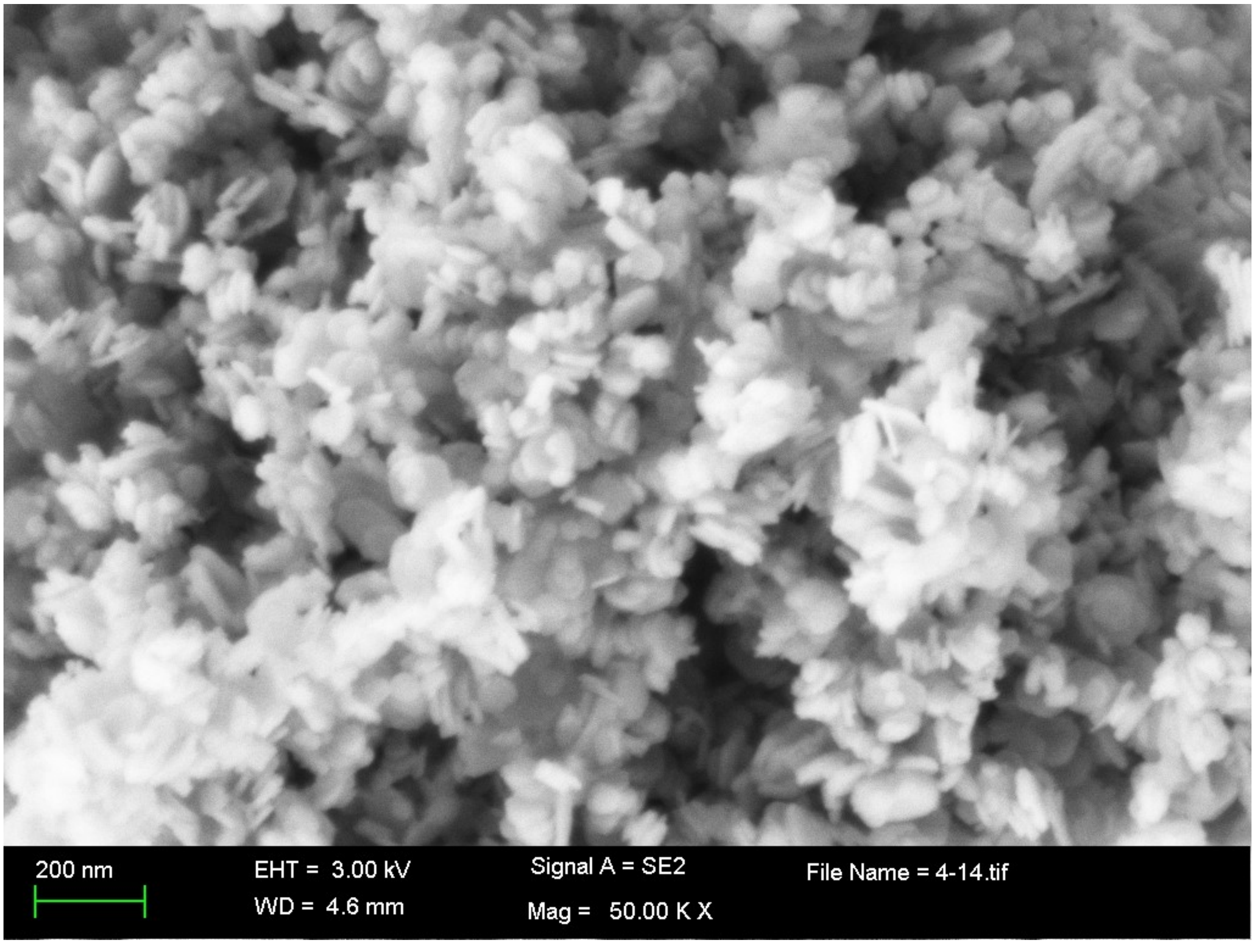
200 nm
EHT = 3.00 kV
WD = 4.6 mm
Signal A = SE2
Mag = 50.00 K X
File Name = 4-14.tif
